# Supplementary material for: Sex specific effects of adoptive Tregs transfer on the brain and periphery in maternal immune activation offspring rescuing immune dysregulation
Source: J Neuroinflammation. 2026 Mar 12;23:133. doi: 10.1186/s12974-026-03739-w (PMC13097898; doi:10.1186/s12974-026-03739-w)
Supplement: Supplementary file 2 — Supplementary Material 2. [file 12974_2026_3739_MOESM2_ESM.docx]

**
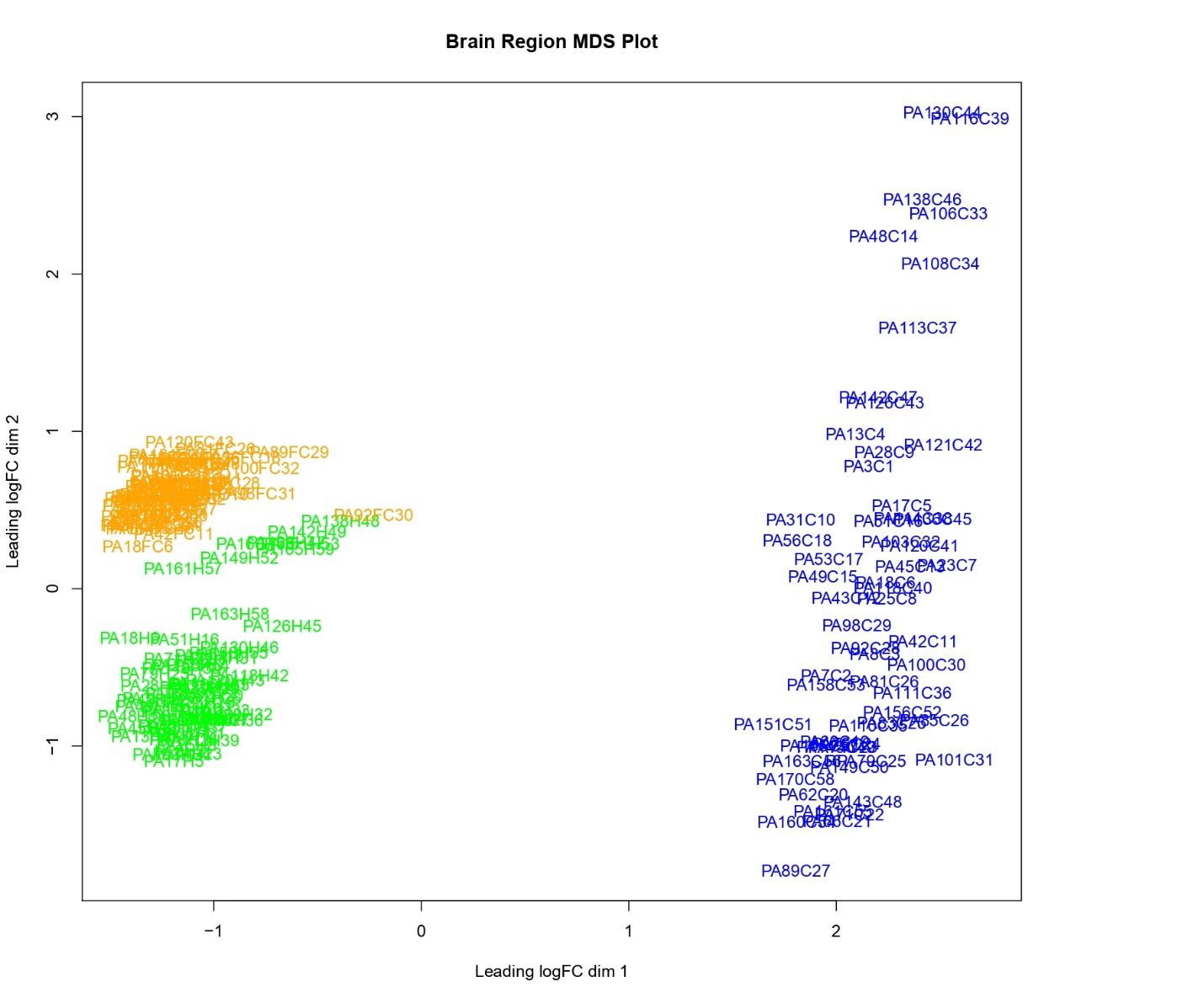
Figure S3** Multidimensional Scaling plots of the top 500 most variably expressed genes revealed that RNA transcriptomes across all study groups and sexes separated along the first two dimensions depending on the brain region.


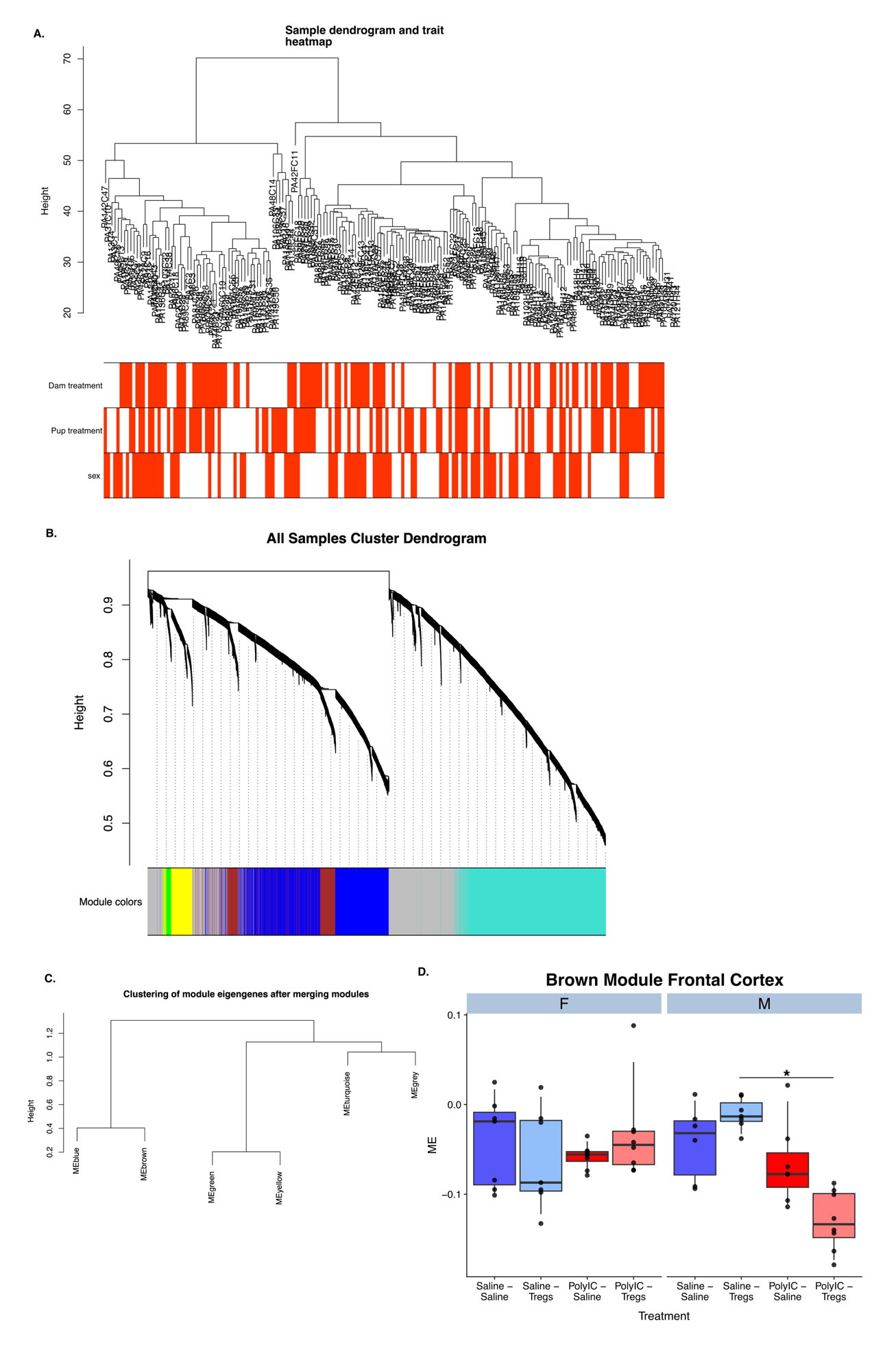


**Figure S4 Weighted gene co-expression network analysis (WGCNA) module identification and eigengene relationships across treatments, sexes, and brain regions.** (**A**) **Sample dendrogram and trait heatmap** showing hierarchical clustering of all samples based on gene expression profiles. Colored bars below the dendrogram indicate experimental traits: dam treatment (Poly I:C vs saline), pup treatment (Tregs vs saline), and sex (male vs female). (**B**) **Gene dendrogram and module assignment** depicting clustering of genes based on topological overlap and subsequent dynamic tree cutting. Modules are shown as color-coded bands below the dendrogram (e.g., blue, turquoise, brown, green, yellow). (**C**) **Module eigengene clustering** illustrating relationships among identified modules based on eigengene correlations, with closely related modules merged prior to final analyses. (**D**) **Brown module eigengene expression in the frontal cortex** stratified by sex (F = female, M = male) and treatment. Boxplots represent module eigengene (ME) values for each group. Asterisks indicate significant post hoc differences between treatment conditions (*p < 0.05, Benjamini–Hochberg corrected).


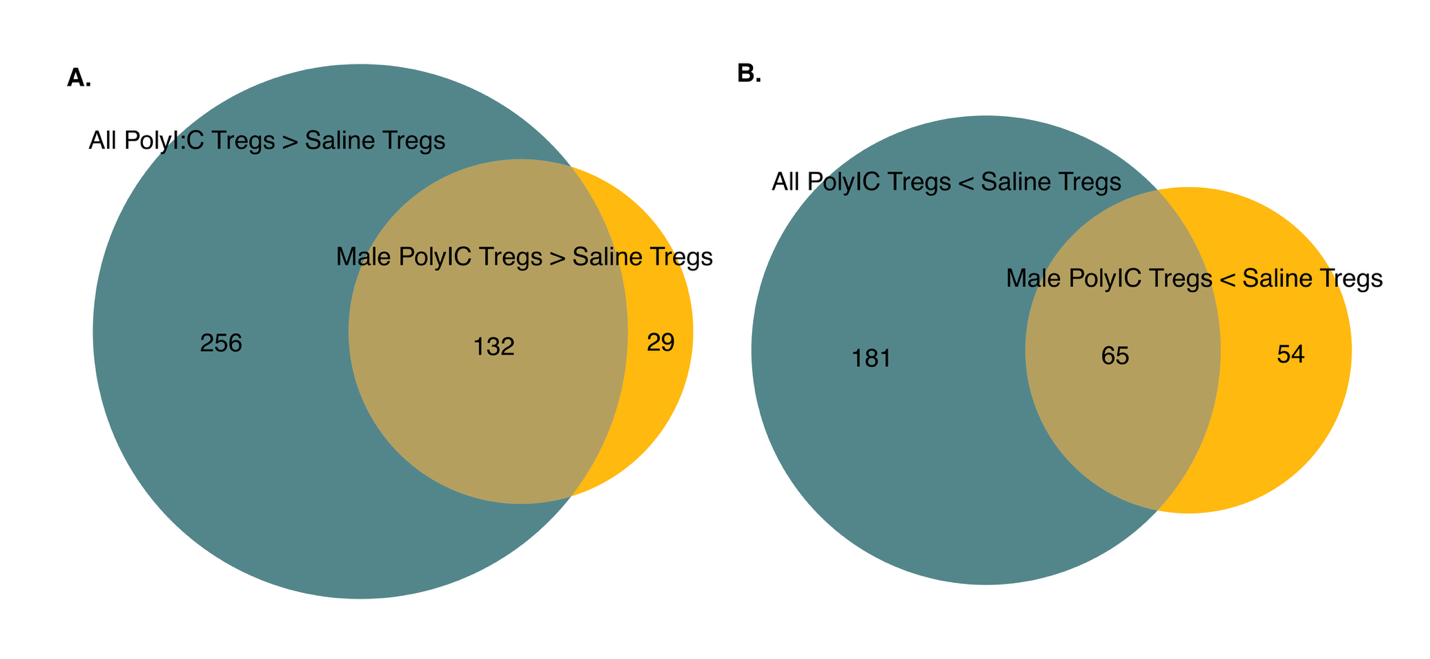


**Figure S5** Genes differentially increased (**A**) and decreased (**B**) in the cerebellum of offspring treated with Tregs whose dam received either Poly I:C or Saline for males (gold) versus both sexes (All).


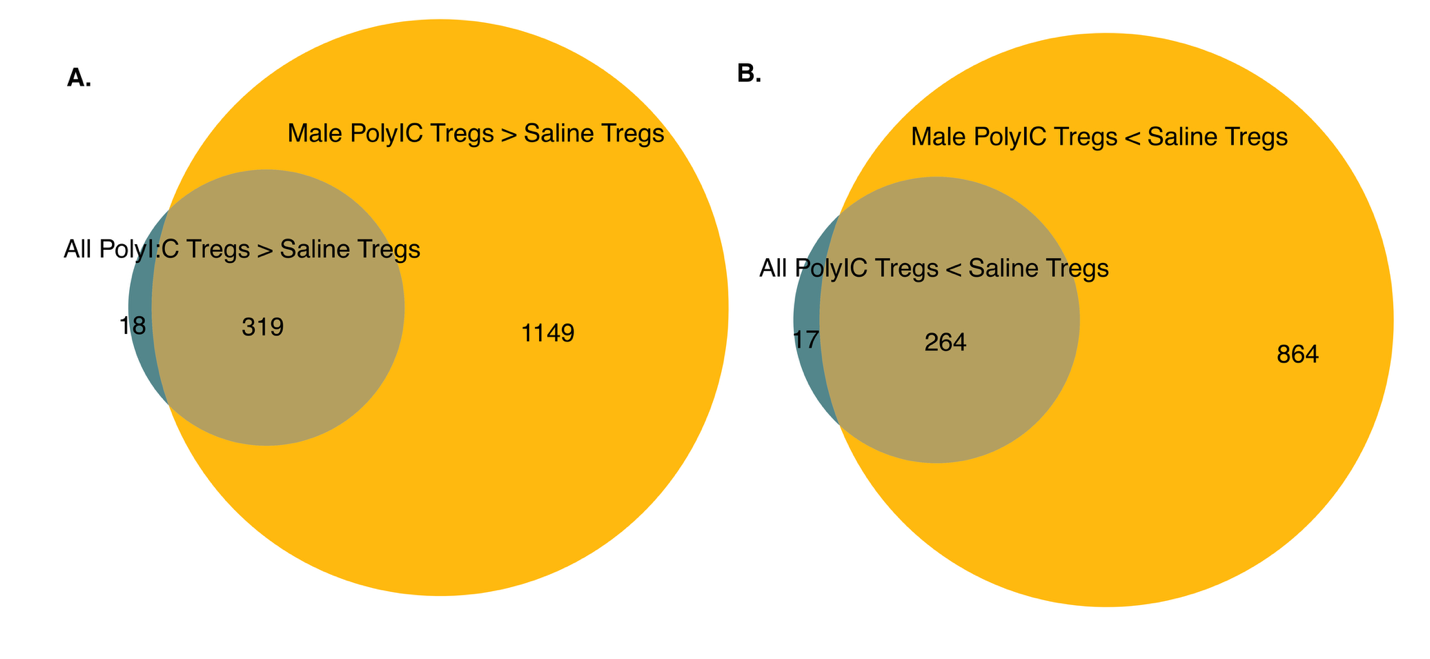
 **Figure S6** Genes differentially increased (**A**) and decreased (**B**) in the frontal cortex of offspring treated with Tregs whose dam received either Poly I:C or Saline for males (gold) versus all both sexes (All).
