# Supplementary material for: Sex specific effects of adoptive Tregs transfer on the brain and periphery in maternal immune activation offspring rescuing immune dysregulation
Source: J Neuroinflammation. 2026 Mar 12;23:133. doi: 10.1186/s12974-026-03739-w (PMC13097898; doi:10.1186/s12974-026-03739-w)
Supplement: Supplementary file 4 — Supplementary Material 4. [file 12974_2026_3739_MOESM4_ESM.docx]

**Table S2** Weighted gene co-expression network analysis (WGCNA) results showing gene modules associated with early-life immune activation and Tregs treatment. This table compiles results from WGCNA analyses integrating module-level statistics, post hoc tests, and gene enrichment data across brain regions and experimental conditions. Each tab contains the following:

· WGCNA Data: Module assignment and eigengene values for all genes included in the co-expression network.

· Module ANOVA Results: Repeated-measures ANOVA results for module eigengenes, with effects of dam treatment, pup treatment, sex, and brain region.

· Module BH posthocs: Benjamini–Hochberg corrected post hoc pairwise comparisons for module eigengenes across experimental groups.

· GO Enrichments: Gene Ontology enrichment results (biological process, cellular component, molecular function) for each significant WGCNA module.

· MGEnrichments: Microglia-specific gene set enrichment analyses identifying cell-type or functionally relevant module associations.

· Top Hub Genes: List of the top hub genes (highest intramodular connectivity) for each module, indicating key drivers of co-expression patterns.
