# Supplementary material for: Sex specific effects of adoptive Tregs transfer on the brain and periphery in maternal immune activation offspring rescuing immune dysregulation
Source: J Neuroinflammation. 2026 Mar 12;23:133. doi: 10.1186/s12974-026-03739-w (PMC13097898; doi:10.1186/s12974-026-03739-w)
Supplement: Supplementary file 5 — Supplementary Material 5. [file 12974_2026_3739_MOESM5_ESM.docx]

**Table S1** Differential gene expression analysis across brain regions following early-life immune activation and Tregs treatment. This table summarizes RNA-seq–derived differential expression results and functional enrichment analyses across the cerebellum, frontal cortex, and hippocampus. Each tab contains the following information:

· Master Experiment Info: Summary of all experimental groups, treatments, sample metadata, and sequencing details.

· DEG counts: Number of differentially expressed genes (DEGs) identified per comparison, including thresholds and region-specific summaries.

· Cerebellum DEGs: Full DEG list for cerebellar samples, including gene identifiers, log₂ fold changes, p-values, and adjusted FDRs.

· Frontal Cortex DEGs: DEG table for frontal cortex samples with statistical outputs and annotation details.

· Hippocampus DEGs: DEG table for hippocampal samples with direction and magnitude of expression changes.

· GO CB DamPolyICvSal_MalePup: Gene Ontology enrichment results for cerebellar DEGs from the dam Poly I:C vs saline comparison in male pups.

· CB DEGs MGEnrichments: Microglia-specific gene set enrichments for cerebellar DEGs.

· GO FC DamPolyICvSal_MalePupTreg: GO enrichment results for frontal cortex DEGs from male pups comparing Tregs vs saline under dam Poly I:C treatment.

· FC DEGs MGEnrichments: Microglia-related enrichment analysis for frontal cortex DEGs.

· GO HC DamSal_MalePupSalvsTreg: GO enrichment analysis for hippocampal DEGs comparing saline vs Tregs treatment in male pups from saline-treated dams.

· HC DEGs MGEnrichments: Microglia gene-set enrichment results for hippocampal DEGs.
