# Supplementary material for: Sex specific effects of adoptive Tregs transfer on the brain and periphery in maternal immune activation offspring rescuing immune dysregulation
Source: J Neuroinflammation. 2026 Mar 12;23:133. doi: 10.1186/s12974-026-03739-w (PMC13097898; doi:10.1186/s12974-026-03739-w)
Supplement: Supplementary file 6 — Supplementary Material 6. [file 12974_2026_3739_MOESM6_ESM.docx]

**
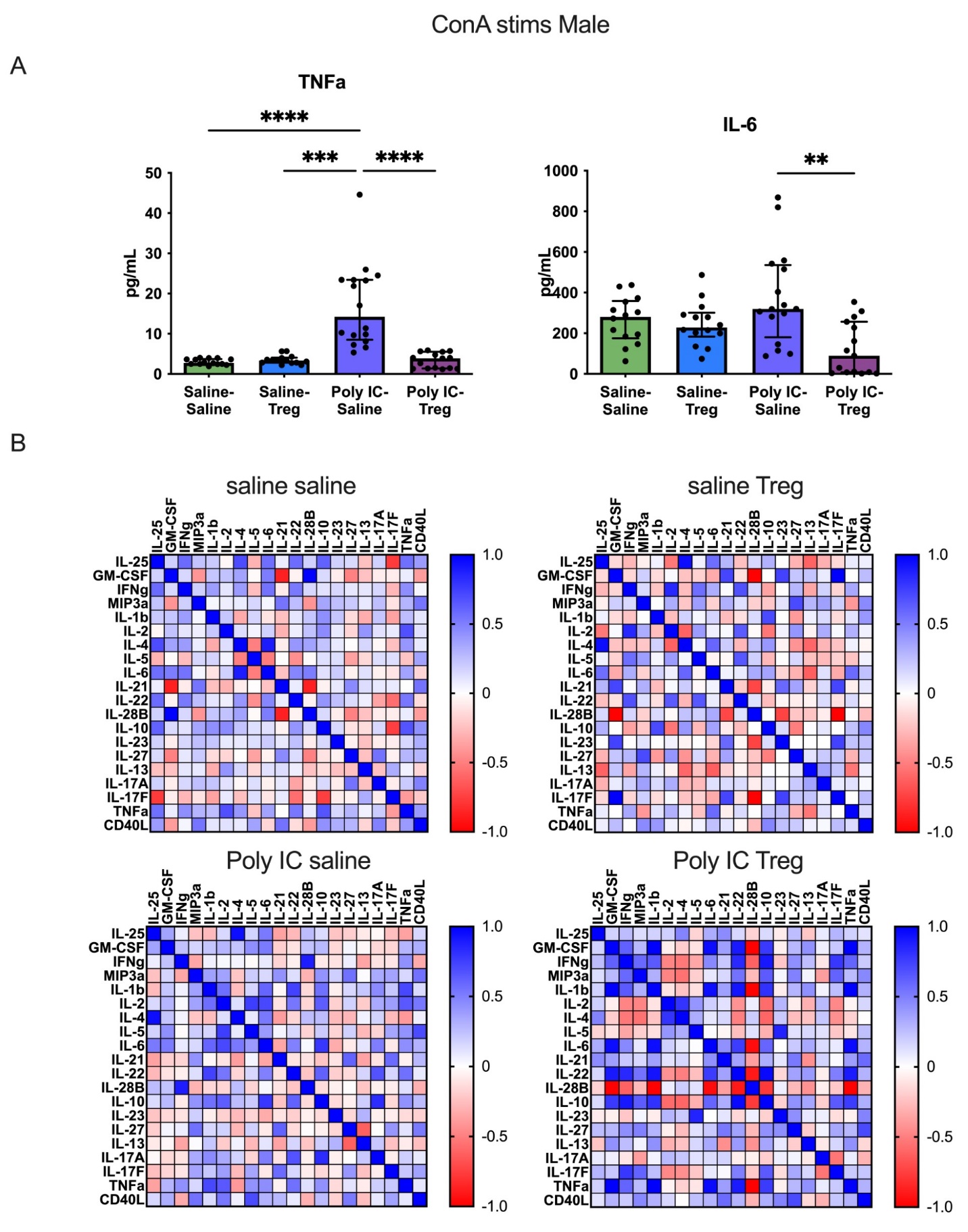
Figure S1** Splenocytes from male offspring were stimulated with Concanavalin A for 48 hours. Cytokines from cell culture supernatant were measured using Multiplex Luminex assays. (**A**)TNFα and IL-6 were significantly downregulated in male Poly I:C-Tregs offspring compared to their Poly I:C-Saline counterparts. (**B**) Cytokine correlations matricies following Con A stimulation in each group. In male Poly I:C-Tregs offspring, several cytokines were strongly correlated, whereas this was not observed in other study groups. P-values are represented as *(<0.05), **(<0.005), ***(<0.0005), **** (<0.00005)
