# Supplementary material for: Sex specific effects of adoptive Tregs transfer on the brain and periphery in maternal immune activation offspring rescuing immune dysregulation
Source: J Neuroinflammation. 2026 Mar 12;23:133. doi: 10.1186/s12974-026-03739-w (PMC13097898; doi:10.1186/s12974-026-03739-w)
Supplement: Supplementary file 7 — Supplementary Material 7. [file 12974_2026_3739_MOESM7_ESM.docx]

**Figure S2** Splenocytes from female offspring were stimulated with Concanavalin A for 48 hours. Cytokines from cell culture supernatant were measured using Multiplex Luminex assays. (**A**) IL-25, IL-6 and IL-2 were significantly different across various study groups. (**B**) Cytokine correlations in female Saline-Saline, Saline-Tregs, and Poly I:C-Saline study groups all had strong cytokine production that correlated with each other. However, in female Poly I:C-Tregs offspring, many of these strong correlations were not observed. P-values are represented as *(<0.05), **(<0.005), ***(<0.0005)
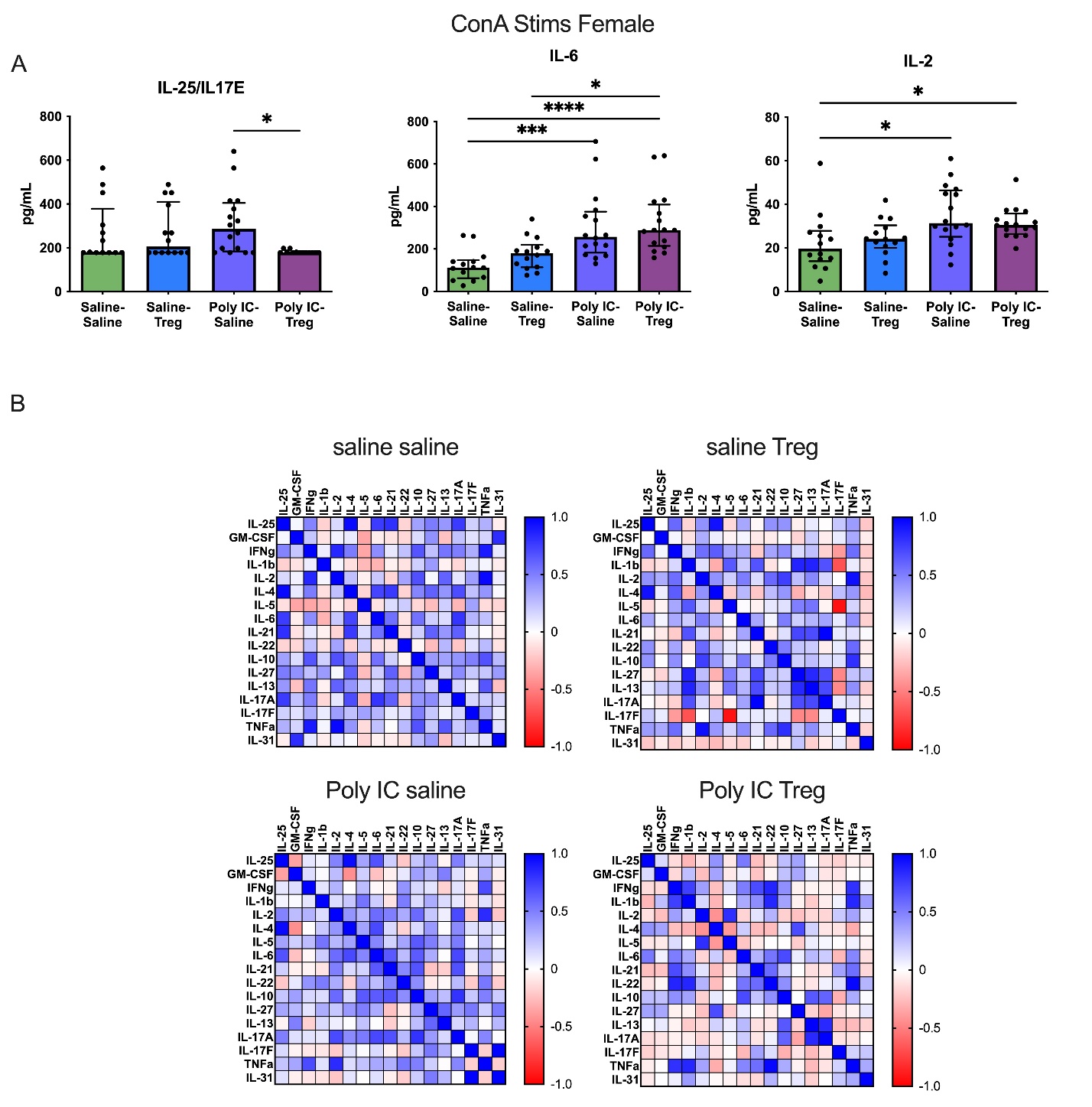
, ****(<0.00005).
