# Supplementary material for: Sex specific effects of adoptive Tregs transfer on the brain and periphery in maternal immune activation offspring rescuing immune dysregulation
Source: J Neuroinflammation. 2026 Mar 12;23:133. doi: 10.1186/s12974-026-03739-w (PMC13097898; doi:10.1186/s12974-026-03739-w)
Supplement: Supplementary file 8 — Supplementary Material 8. [file 12974_2026_3739_MOESM8_ESM.docx]

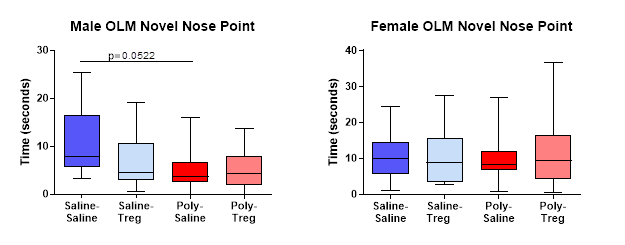


Supplemental Figure 5 - Object Location Memory Tests. To access the long-term and spatial memory across our study groups, we utilized tasks evoking object location memory (OLM)(Vogel-Ciernia & Wood 2015). Mice were habituated to clean, empty plexiglass cages for 5 minutes a day for 6 days. Mice are then trained with two objects. After 24 hours, mice were placed back into the test cage, with one object removed to a new location. The amount of time spent with the object in the novel location is measured, with greater time spent with the object in the new location indicative of greater OLM. Significant differences were determined using 2-Way ANOVAs accounting for dam, offspring and dam x offspring interactions. P-values are represented as *p < 0.05.
